# 

**Published:** 1878-07

**Authors:** 


### CONTENTS.

PAGE.

**CONTRIBUTIONS.**

The Case of Brown. Ben. H. Pratt ....... 31
The Game of the Right Hand Against the Left. P. Harold Hayes, M. D. ....... 34
Success in Life. Ausburn Towner ....... 35
How Shall we Train Our Boys. Uncle Bob ....... 38

**MEDICAL ITEMS AND NEWS.**

Deodorized Iodoform—Sclerotinic Acid for Hypodermic Injection—Pepsine from Ostrich—Laboratory Vintage—Arsenical Poisoning—Chorea—Prurigo — Painful Hemorrhoids — Dysentery—Articular Rheumatism — Chloral — Whooping Cough — Hemorrhoids — Electuarium Sennæ Compositum—Fuller's Tamarind Electuary—Incontinence of Urine—Sedative Pills—Lotion for Sore Nipples Abscess of Liver—Neutralizing Poison with Sweet Oil—Bossu's Laxative mixture—Arsenate of Gold—Dialysed Iron Hypodermically—Hoarseness—Diabetes Insipidus—Aromatic Licorice—Eruption by Poison Oak—R. Rother's Bay Rum Formula—Chronic Pleurisy—Ergotine Suppositories—Soluble Medicated Intra-uterine Bougies—Chloral and Opium—Local Antiseptic—Delirium Tremens—Sulphuric Acid Beer— Cod Liver Oil with Hypohosphites and Whiskey—Use of Ergotine in Uterine Fibroids—Epistaxis—Elimination of Mercury — Typhoid Fever — Ingrowing Toe Nail — Rheumatism — Quinetum—Oil Bath in Disease—Burns ....... 40 to 47

PAGE.

**OUR CORRESPONDENCE.**

Letter from London—Letters on Horseback... 48 and 49

**HOUSEHOLD HINTS AND RECIPES.**

Table Salt in Milk for Children—Promote Growth of Whiskers—Tea—Hair Tonic—Lemon Cordial—Liquid Shoe Varnish—Clean Paint—Preserve Fruits—Test for Milk—Detection of Artificial Butter—Uses of Lemon—Black Ink—Artificial Flower Barometers—Glue for Damp Places—To Mend India-rubber—Preventive of Lead Poisoning—Polish Silver—Green Ink ....... 50 and 51

**EDITORIAL.**

Home Treatment of Eyes ....... 52
Children's Legs ....... 25
Summering ....... 53

**EDITORIAL CHIT-CHAT.**

Fourth of July—A Little Late—The Score—Ausburn Towner—Which?—Our Schools—Bound Bistourys—Our Vacation—The Paragraphers—Seeds—Our Boys and Girls—A Threatened Divorce—Responses—A Total Wreck—Roosting—June—A Queer Machine—Will C. Potter—Mean Men—That Woodchuck—The Ever-glorious—Buggy ....... 56 to 58

